# Fabrication of Micro Carbon Mold for Glass-Based Micro Hole Array

**DOI:** 10.3390/mi15020194

**Published:** 2024-01-27

**Authors:** Ui Seok Lee, Dae Bo Sim, Ji Hyo Lee, Bo Hyun Kim

**Affiliations:** 1 Department of Mechanical Engineering, Graduate School, Soongsil University, 369 Sangdo-ro, Dongjak-gu, Seoul 06978, Republic of Korea; uslee@prelab.ssu.ac.kr (U.S.L.); daebosim@soongsil.ac.kr (D.B.S.); jihyolee@soongsil.ac.kr (J.H.L.); 2 School of Mechanical Engineering, Soongsil University, 369 Sangdo-ro, Dongjak-gu, Seoul 06978, Republic of Korea

**Keywords:** micro hole array, micro mold, eccentric tool, glass-based biochip, glass molding

## Abstract

In glass molding to produce biochips with micro holes, cavities, and channels, it is important to machine micro molds. This study presents a novel process for fabricating micro pin arrays on carbon graphite, one of the glass molding materials. The micro pin array was used as a mold to fabricate a glass-based micro hole array. Using conventional micro endmill tools, machining micro-cylindrical pins requires complex toolpaths and is time-consuming. In order to machine micro pin arrays with high efficiency, a micro eccentric tool was introduced. Micro pin arrays with a diameter of 200 µm and a height of 200 µm were easily fabricated on graphite using the micro eccentric tool. In the machining of micro pin arrays using eccentric tools, the machining characteristics such as cutting force and tool wear were investigated.

## 1. Introduction

Micro hole arrays or micro well arrays enable the analysis of hundreds to thousands of cells at the single-cell level, with applications in the biomedical industry, including cell biology and tissue engineering [1]. Micro hole arrays have been employed as powerful tools for live cell imaging, manipulation of cell–cell interactions, and cell culture [2]. The size, shape, and geometry of the micro holes vary depending on the function and application, which can range from a few micrometers to several hundred micrometers.

Polydimethylsiloxane (PDMS) polymers are most commonly used as the material of micro hole arrays due to their advantages, including low toxicity, desirable optical transparency, and biocompatibility [3]. However, it has the disadvantages of low mechanical strength and low chemical stability [4].

Glass is one of the preferred materials for microchips in the biomedical industry because it has high transparency, high chemical resistance, inertness to most substances, and ability to withstand higher temperatures compared to polymer materials [5]. Due to the brittleness of glass, however, brittle fracture or severe surface cracks can easily occur during machining. As a result, micro machining of glass is still a challenge, and various methods of machining micro holes or micro patterns in glass have been studied [6].

As methods for micro machining of glass, dry/wet etching, mechanical drilling, abrasive jet machining, electrochemical discharge machining (ECDM), and laser processing, etc., can be considered.

Wet etching using hydrofluoric acid (HF) is the most common and well-developed method for glass micro machining. The etching rate is fast, and large quantities of glass chips can be produced at a reasonable cost. However, there are limitations in making precise microscale profiles due to isotropic etching and undercut in glass machining [5]. In contrast, dry etching methods such as reactive ion etching (RIE) can create anisotropic profiles. However, this method has the disadvantage of requiring a very long etching time for glass, and a high cost [7].

Mechanical drilling can produce micro hole arrays at low cost. Since material is removed by mechanical forces, micro tools can be easily broken, and cracks can occur at the entrance or exit of the hole. Therefore, ultrasonic vibration is often applied to the glass during drilling. Additionally, tool materials with much higher hardness than glass, such as diamond are required for high-quality hole drilling. Kim et al. reported on microdrilling of glass using diamond and carbide drills [8]. Egashira et al. applied 40 kHz ultrasonic vibration while drilling glass, and drilled micro holes with a diameter of 10 µm on borosilicate glass [9]. Cheng et al. used polycrystalline diamond (PCD) drilling tools and showed that high-quality small hole drilling in glass could be achieved with extremely little brittle fracture and cracking on the surface [10]. Schorderet et al. used ultrasonic vibration to machine micro holes with a diameter of 100 µm and a depth of 1000 mm [11].

In abrasive jet machining or powder blasting, abrasive particles are injected through a nozzle into glass at very high speeds. Since material is rapidly removed by the kinetic energy of the abrasive particles, this method shows a very high machining rate. Suresh et al. drilled micro holes on borosilicate glass and investigated the effect of pressure and particle size of abrasives on the material removal rate [12]. Wensink et al. proposed powder blasting for fabricating micro channels with an aspect ratio of 2.5 on glass [13]. Schwartzentruber et al. used abrasive waterjet machining to drill micro holes, which uses high pressure water [14]. In the machining of very small features, however, there are limitations to the size of the abrasive particles and masks. Since a material is removed due to the collision of abrasive particles, cracks may remain on the surface, and it is difficult to control the machining depth precisely.

Yen et al. used a CO_2_ laser to fabricate microfluidic chips on Borofloat and Pyrex glass [15]. Kuriakose et al. drilled micro through-holes on sapphire using a Ti:Sapphire amplified laser [16]. They used ultrashort Bessel beams to fabricate micro holes with a negligible crack. Zhou et al. applied a femtosecond laser to damage and modify chalcogenide glass, and then used wet etching to fabricate micro lens arrays with excellent morphology and surface quality [17]. Chemical and thermal methods such as ECDM can be used for micro machining of glass. Bhargav et al. studied the fabrication of micro holes with a thickness of 1 mm in borosilicate glass using an electrolyte-sonicated micro ECDM system [18]. However, methods using CO_2_ lasers or ECDM can create a heat-affected zone (HAZ), which can negatively affect surface quality and dimensional accuracy. Additionally, an ultrashort laser for glass machining is very expensive and is not suitable for mass production.

On the other hand, glass molding is a suitable method for mass producing glass-based products at low cost and high productivity. Many researchers have studied glass molding for fabricating microchips and micro hole arrays [7,19,20,21]. In glass molding, tungsten carbide and silicon carbide have been used as mold materials. However, these materials have high hardness (HRC > 90), making it difficult to machine micro molds [21]. Graphite is another glass molding material that is relatively easy to machine. It has high enough stiffness and heat-resistance to be a mold material [22,23,24]. Since it has high brittleness, it does not generate much burr during mechanical machining.

Glass molding is highly productive, but machining molds with many micro features requires complex tool paths and is time consuming. In order to improve machining efficiency, in this study, a novel micro eccentric tool was proposed to machine a large number of micro pins on graphite material. The pins were used as molds to produce micro hole array chips by glass molding. This study presents the fabrication process of micro eccentric tools using electrical discharge machining (EDM). To confirm the feasibility of micro eccentric tools, the machining characteristics were investigated. Thrust force was measured according to feedrate and spindle speed, and tool wear was analyzed.

## 2. Materials and Methods

### 2.1. Experimental Setup

**Figure 1:**
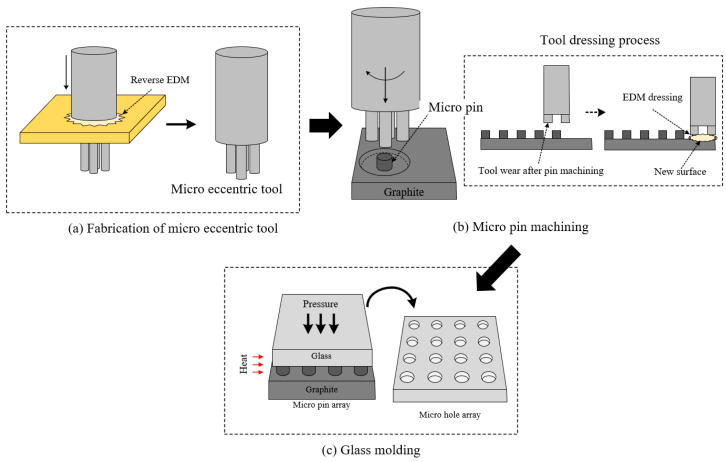
Fabrication process of glass-based micro hole array using eccentric tools.

Figure 1 shows the overall process for fabricating glass-based micro hole arrays. First, an eccentric tool is fabricated using reverse EDM (Figure 1a). The detailed process for the tool fabrication is described in the next section. Next, a large number of micro pins are machined on a graphite plate using the eccentric tool (Figure 1b). When the tool wears out during pin machining, the tool surface is regenerated by EDM dressing. Then, the micro hole array is fabricated through glass molding using the micro pin array as a mold (Figure 1c).

**Figure 2:**
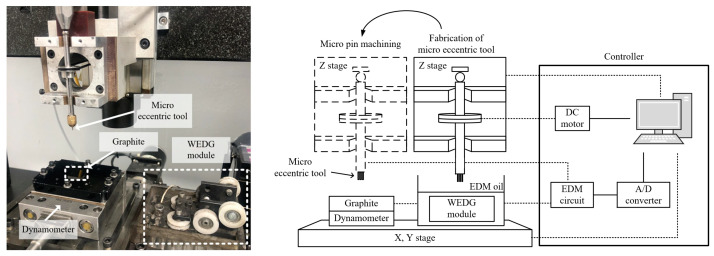
Experimental setup (both micro tool fabrication and micro pin array machining were performed on the same system).

Figure 2 shows a photo and a schematic diagram of the experimental system for machining the eccentric tools and micro pin arrays on the graphite plate. The system includes a mandrel spindle system, 3-axis positioning stages, a dynamometer for force measurement, a WEDG (wire electro discharge grinding) module for micro tool fabrication, and an EDM pulse generator [25]. Both micro tool fabrication and micro pin array machining were performed on the same system.

WEDG is one of the widely used methods for micro tool fabrication. It is a variant of wire EDM that can easily produce high aspect ratio micro tools with diameters of tens of microns [26]. The WEDG module consists of an EDM bath, a wire guide, and several pulleys. The tool material is machined by electrical discharge generated between the tool electrode and a brass wire with a diameter of 200 µm. The wire continues to move along the pulley and wire guide, so wire wear is negligible during machining. An RC type circuit was used as the EDM pulse generator. Kerosene was used as a dielectric fluid in EDM and as a cutting fluid in the micro pin array machining. The micro pin machining was performed in a bath filled with the kerosene. The kerosene was useful to flush away the chip between the tool and the workpiece. The mandrel spindle system consists of a mandrel, V-block, and a servo motor mounted on a Z-axis stage. The mandrel, driven by a servo motor and a belt pulley, holds a tungsten carbide rod with a diameter of 1 mm. The maximum tool rotational speed was 4500 rpm. The workpiece was 95% high-purity carbon graphite with a size of 15 mm × 15 mm and a thickness of 1 mm. The dynamometer (9256C2, Kistler Instrumente AG, Winterthur, Switzerland) was installed under the graphite workpiece to measure the forces as the micro pin array was machined. The surface roughness of a tool bottom was measured by a laser confocal microscope (OLS5000, Olympus Corp., Tokyo, Japan).

### 2.2. Fabrication of Micro Eccentric Tool

A micro eccentric tool was fabricated by micro EDM. In EDM, material is removed by electrical sparks generated between a tool electrode and a workpiece. Thus, the micro EDM has advantages of machining any electrically conductive materials regardless of hardness and of machining complicated shapes with high accuracy [26].

Tungsten carbide (WC) was used as the material for the micro eccentric tool and micro EDM drilling tool. Tungsten carbide is characterized by high hardness, strength, and wear resistance over a wide temperature range [27]. Due to these properties, tungsten carbide is a difficult-to-machine material by conventional processes.

**Figure 3:**
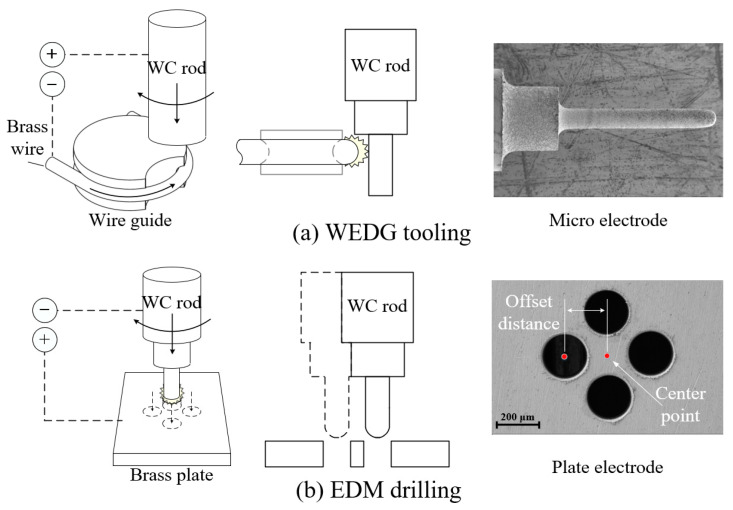

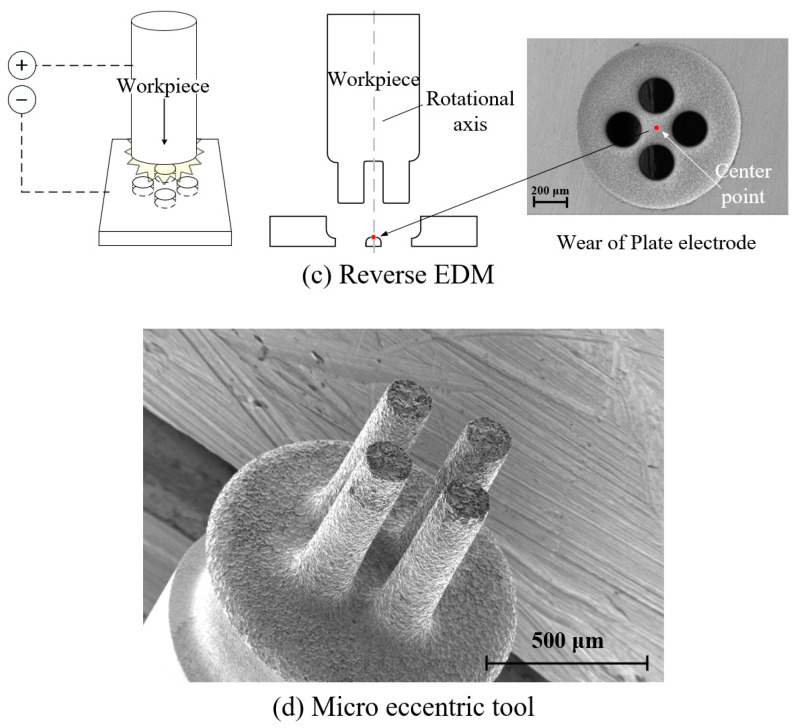
(**a**–**c**) schematic diagram of eccentric tool fabrication process and (**d**) SEM image of micro eccentric tool.

Figure 3 shows the machining process of micro eccentric tools. First, a WC rod with a diameter of 1 mm was machined into a micro tool electrode by WEDG, as shown in Figure 3a. Table 1 shows the machining parameters. A voltage of 100 V was applied using a DC power supply by connecting the anode to the WC rod and the cathode to the brass wire.

A capacitance of 20,000 pF was used because the higher the capacitance, the rougher the surface of the tool, which is effective for mold machining. The feedrate of the WC rod was 5 µm/s, and the rotational speed was 2500 rpm.

Considering the tool wear during micro EDM drilling, a micro electrode with a length of 1.5 mm and a diameter of 194 µm was fabricated. In EDM, discharge occurred through the gap between the tool and the workpiece. The discharge gap distance varies depending on the applied voltage, capacitance, and tool size. Under the discharge conditions in this study, it was approximately 3 µm. The tool size was determined by considering the accurate hole size based on the discharge gap.

The micro tool electrode was used to drill four micro holes with a diameter of 200 µm on a brass plate by micro EDM, as shown in Figure 3b. It is noted that the position of each hole had the same offset distance and were symmetrical about the center point. As shown in Figure 3c, another WC rod was replaced while maintaining the position of the plate. Since the spindle system used the V-block and the mandrel, it was very easy to replace the WC rod. The brass plate was used as a plate electrode for reverse EDM [28]. The rod (anode) was fed to the plate electrode (cathode). Then, four micro eccentrical tools with eccentricity around the center point were machined as shown in Figure 3d.

### 2.3. Micro Pin Machining by Eccentric Tool

**Figure 4:**
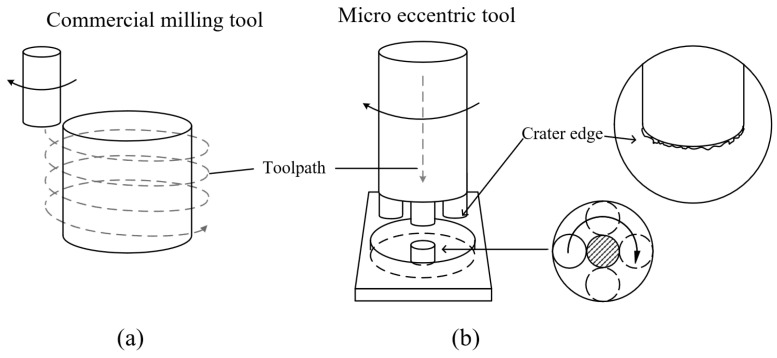
Comparison between the toolpath of (**a**) a commercial milling tool and (**b**) a micro eccentric tool.

Figure 4 shows the comparison of micro pin machining by a conventional end mill tool and micro eccentric tool. In conventional micro milling shown in Figure 4a, precise X-Y-Z directional motion control of the tool along complicated toolpaths is required. Because the tool diameter is small, high tool rotational speed is needed to acquire high cutting speed. With the micro eccentric tool, however, the toolpath is very simple to generate micro pins; it has only vertical motion such as a drilling process, as shown in Figure 4b. The material removal mechanism of the micro eccentric tool is different from that of the conventional micro milling tool. Since the micro eccentric tool was fabricated by EDM, the tool bottom has many discharge craters on its surface. During the machining of micro pins, the edge of a crater on the micro eccentric tool surface works as an abrasive grit, as in a grinding process. A graphite workpiece is removed by the craters on the tool bottom surface as shown in Figure 4b.

## 3. Results

The machining characteristics according to machining conditions were investigated in micro pin machining using micro eccentric tools. Thrust forces were measured according to tool feedrate, tool rotation speed, and tool bottom roughness. The tool wear and tool dressing were also investigated. The machining conditions are shown in Table 2.

### 3.1. Effects of Tool Axial Feedrate and Rotational Speed

**Figure 5:**
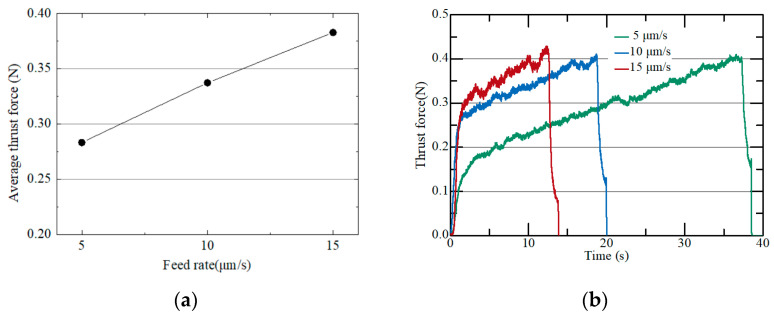
(**a**) Average thrust force according to different feed rates; (**b**) thrust force measured in a single pin machining (4500 rpm; depth: 200 µm).

In order to investigate the effects of axial feedrate and rotational speed of a tool on grinding force, the thrust force was measured. Figure 5a shows thrust force at different axial feedrates of the micro eccentric tool. The tool rotation speed was fixed at 4500 rpm. As the axial feedrate increased from 5 µm/s to 15 µm/s, the average thrust force increased linearly from 0.28 N to 0.38 N. Figure 5b shows the thrust force obtained during micro pin machining. As the machining time increased, the thrust force gradually increased. This is because as the machining depth increased, the graphite chips become more difficult to flush away.

**Figure 6:**
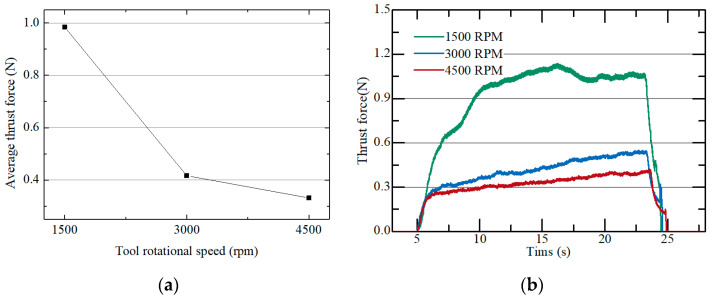
(**a**) Average thrust force according to different tool rotational speeds; (**b**) thrust force measured in a single pin machining (10 µm/s: depth: 200 µm).

Figure 6a shows the thrust force for different rotational speeds of the tool. The axial federate was set at 10 µm/s. Figure 6b shows the thrust force obtained during micro pin machining. As the tool rotation speed linearly increased from 1500 to 3000 and 4500 rpm, the thrust force decreased from 0.985 to 0.416 and 0.332 N. When the tool rotation speed increased from 1500 to 3000 rpm, the thrust force decreased sharply by 57%. However, when the rotation speed increased from 3000 to 4500 rpm, the force slowly decreased by 20%. The tool feed per revolution according to the rotation speed is 400 nm/rev (1500 rpm), 200 nm/rev (3000 rpm), and 133 nm/rev (4500 rpm), respectively. When the feed per revolution is extremely small, the ploughing effect dominates in the material removal process [29]. Therefore, the thrust force does not decrease in proportion to the feed per revolution. Additionally, because the micro tool can break at high thrust force, it is desirable to set the tool rotation speed to 3000 rpm or more.

### 3.2. Effect of Surface Roughness of the Tool Bottom

The effect of surface roughness of the tool bottom on the grinding force was investigated. When using an RC type circuit as the EDM circuit, the discharge energy is proportional to the applied voltage and capacitance, so increasing the capacitance results in a rougher tool surface [30,31]. The EDMed surface consists of many overlapping discharge craters. As a result, the size and shape of the craters affect the surface roughness. The re-solidified layer of the crater also affects the surface roughness. The material removal mechanism of EDM is that the workpiece material is melted by thermal energy, and the molten material flows out by an explosion of dielectric fluid. However, the amount of material that can flow out is limited. As a result, the molten material that fails to flow remains on the machined surface and, as it cools, a resolidification layer is formed. Therefore, as the discharge energy increases, the amount of molten material that cannot flow increases, and the amount and thickness of the resolidified layer also increase. This increases surface roughness.

**Figure 7:**
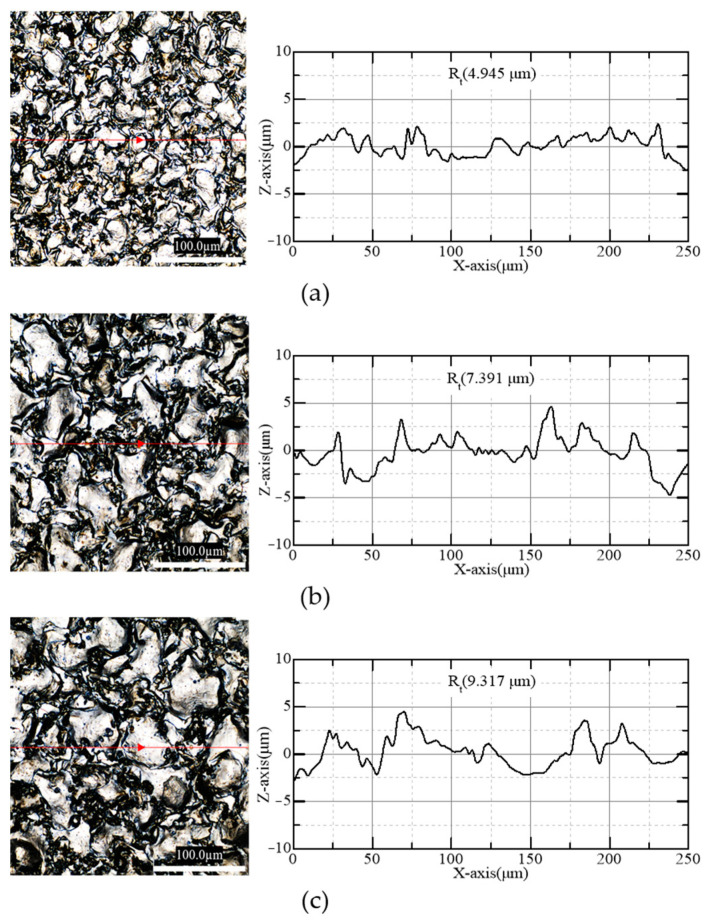
Microscope images and surface profiles of tool bottom surfaces machined with capacitances of (**a**) 50, (**b**) 300, and (**c**) 500 nF.

As shown in Figure 7, the tool bottom surfaces and the surface roughness (Rt, maximum peak to valley height) were measured for capacitances of 50, 300, and 500 nF, respectively. Capacitance has a significant effect on EDM crater size and tool surface roughness [25]. As shown in Figure 7, larger capacitance increases not only the size of craters, but also the protrusion increases, which is measured as Rt.

**Figure 8:**
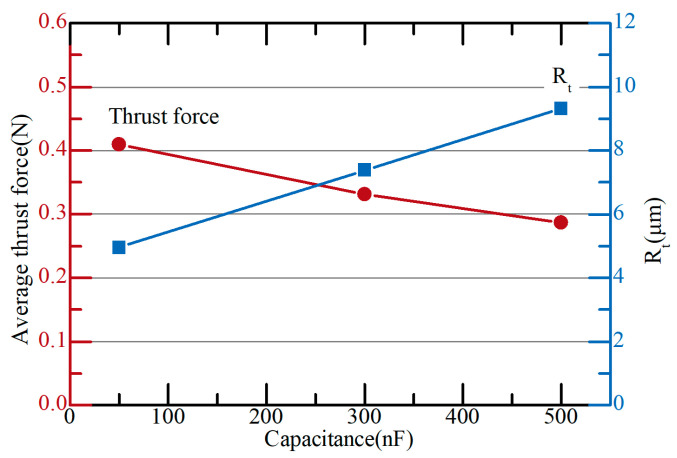
Average thrust force and tool roughness (R_t_) according to different capacitances (10 µm/s; depth: 200 µm; 4500 rpm).

Figure 8 shows the thrust force and surface roughness of the tool according to capacitances. When the capacitance was increased from 50 nF to 500 nF, R_t_ increased from 4.945 µm to 9.317 µm, while force decreased from 0.410 N to 0.287 N. In the grinding process, the greater the surface roughness of the grinding wheel, the lower the grinding force [32]. The craters on micro eccentric tools act like abrasives on a grinding wheel, so as the surface of the tool becomes rougher, thrust force is reduced.

### 3.3. Tool Wear

**Figure 9:**
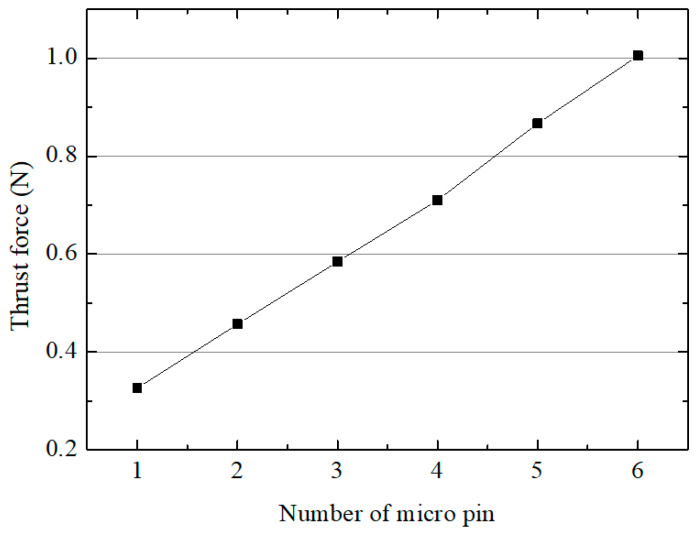
The thrust force according to number of pins (10 µm/s; depth: 200 µm; 4500 rpm).

Tool wear is one of the important issues in the micro machining. In grinding using eccentric tools, tool wear increases grinding forces and shortens tool life [33,34]. As the number of machined pins increases, tool wear and grinding forces increase. Figure 9 shows that the thrust force increased as the number of machined micro pins increased. When a micro pin was machined with the new eccentric tool, the thrust force was 0.325 N. When the sixth pin was machined, however, the thrust force increased to 1 N. This is because wear occurred on the bottom surface of the tool, and the cutting force increased.

**Figure 10:**
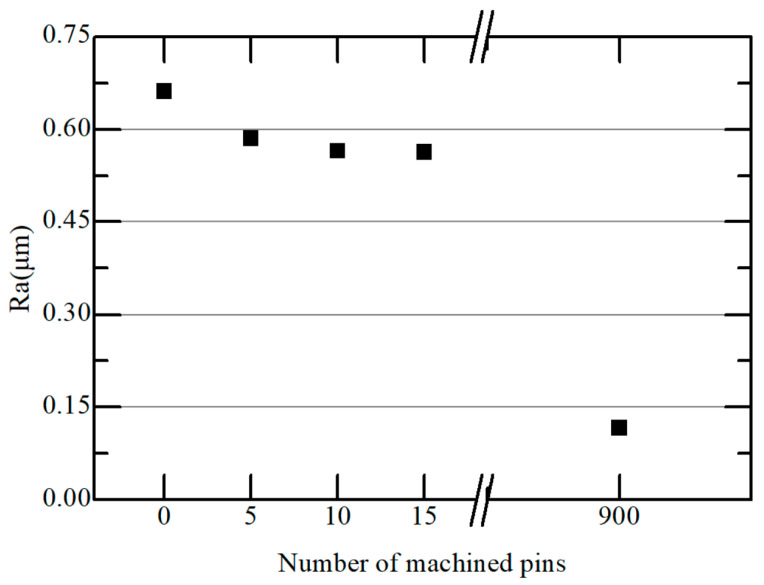
Surface roughness of tool bottom according to the number of machined pins.

**Figure 11:**
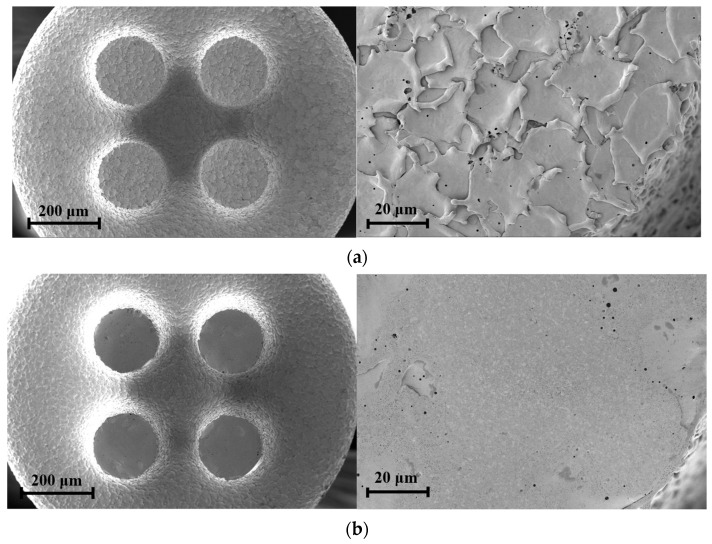
Bottom surface of micro eccentric tool (**a**) before machining and (**b**) after machining 900 pins.

The longitudinal wear of micro eccentric tools was very small and difficult to measure. Therefore, wear was estimated indirectly by the change of surface roughness of the tool bottom. Figure 10 shows the surface roughness (Ra) of the tool bottom according to the number of machined micro pins. When the number of machined pins was 15, Ra was reduced from 0.662 µm to 0.563 µm. When the number of machined pins was 900, the tool bottom became very smooth, with Ra of 0.116 µm. It is noted that the thrust force increased as the surface roughness of the tool bottom decreased. This shows that the graphite was machined by the rough surface consisting of craters at the bottom of the tool. Figure 11 shows the SEM images of the bottom of the eccentric tool before and after machining 900 micro pins. The craters on the bottom of the tool were completely removed. Although the bottom surface of the tool became smooth, the material could be machined with the edge of the tool. However, the thrust force became very high, machining became unstable, and tool breakage often occurred.

### 3.4. Tool Dressing

**Figure 12:**
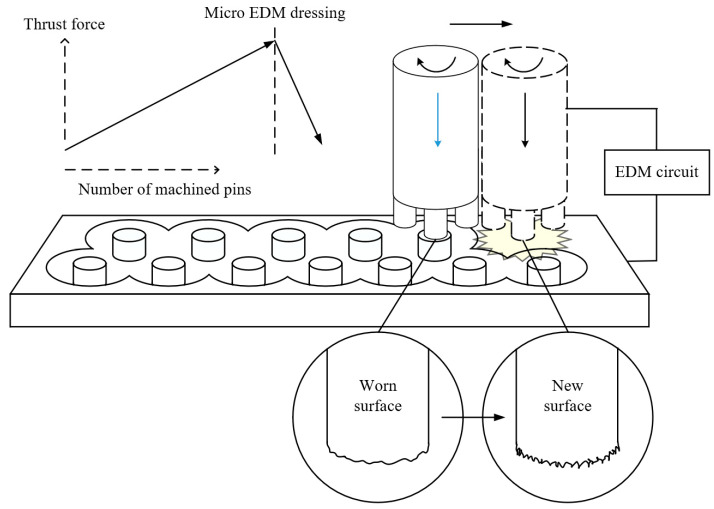
Schematic diagram of EDM dressing process.

**Figure 13:**
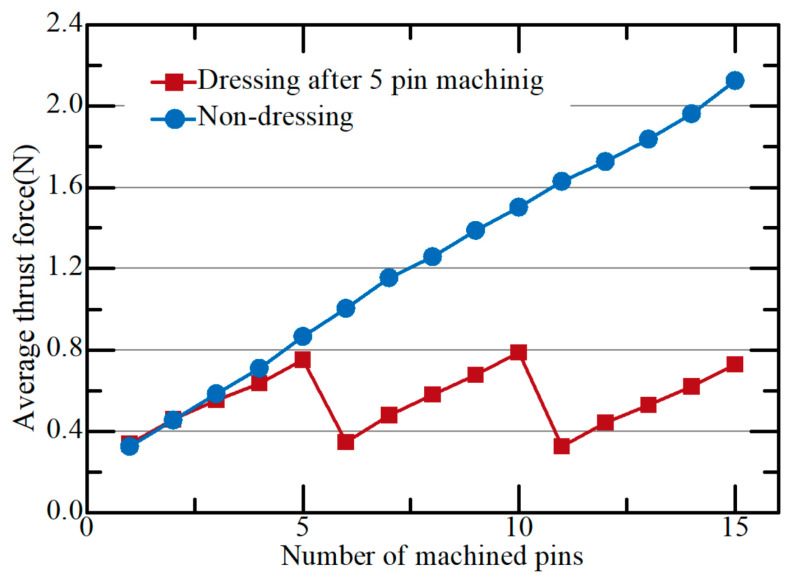
Average thrust force with dressing according to the number of machined pins; 200 µm depths, 4500 rpm, 10 µm/s feedrate.

Tool wear increases thrust force and can result in tool breakage. Therefore, a tool dressing process is necessary to reduce thrust force and extend tool life. In this study, we attempted to regenerate the tool surface by introducing micro EDM dressing. Figure 12 shows a schematic diagram of the micro EDM dressing process of the micro eccentric tool. In micro EDM dressing, the bottom surface of the tool is machined by applying a discharge voltage to the worn tool and graphite plate to generate electrical discharges. The tool bottom was machined to a depth of 5 µm and took less than 5 s. EDM dressing conditions are shown in Table 3. Figure 13 shows the thrust force according to the number of machined pins. Micro EDM dressing was performed after machining every five pins. After dressing, the worn tool surface was sharpened back to the initial rough surface and the thrust force decreased from 0.753 N to 0.357 N. In the machining without dressing, the thrust force continued to increase.

### 3.5. Micro Pin Array for Molding

**Figure 14:**
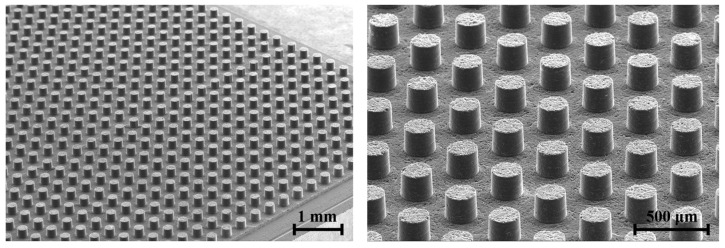
Micro pin array on graphite carbon machined by eccentric tool.

A micro pin array was fabricated on a graphite plate using the micro eccentric tool with an axial feedrate of 10 µm/s and a tool rotational speed of 4500 rpm. Figure 14 shows the array of 900 (30 × 30) micro pins with a diameter of 200 µm and a height of 200 µm. It took 22 s for the machining of each micro pin. The micro pin diameter and height could be machined up to 150 µm and 300 µm, respectively. Smaller sizes and higher aspect ratios of micro pins require process optimization.

**Figure 15:**
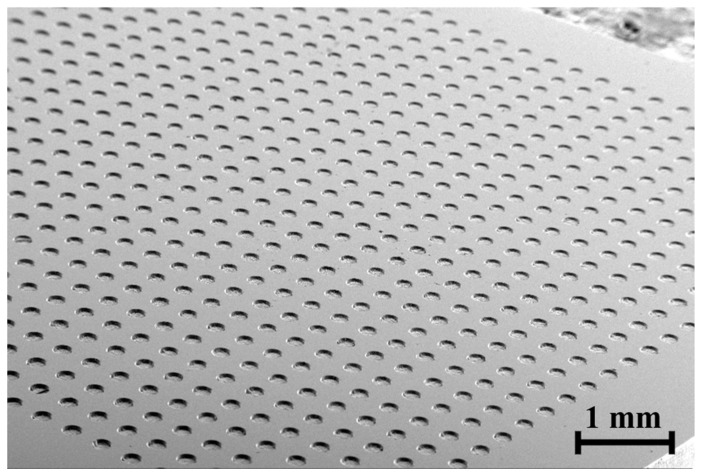
Micro hole array on glass plate by glass molding (hole diameter: 200 µm).

Because glass molding transfers the shape of the mold surface to the glass workpiece, the molding process enables economical mass production of micro parts with micro holes or wells. Figure 15 shows the micro hole array on a glass plate that was fabricated by the glass molding process using the graphite mold. The diameter of the hole was 200 µm and the depth was 50 µm. The thickness of the glass was 0.5 mm. The top surface of the glass was polished for a better surface finish after the molding. The maximum aspect ratio of a micro hole machined by the glass molding is about 0.5, but becomes smaller due to the polishing of the glass surface. During glass molding, the molding temperature was about 250–400 degrees depending on the number or size of micro pins. Usually, graphite molds can be used about 5000 times. However, as the number of pins increases, resistance to demolding increases and the probability of pin breakage increases.

## 4. Conclusions

In this paper, a micro eccentric tool by reverse EDM was introduced to fabricate micro pin arrays on graphite workpieces. Machining a graphite mold with a large number of micro features using a commercial micro end mill tool is very time-consuming. Unlike micro end mill tools, the micro eccentric tool makes micro pins very easy to machine. Machining characteristics using a micro eccentric tool were investigated according to the axial feedrate, rotational speed, and surface roughness of the tool. Additionally, the effect of the number of pins machined on tool wear and thrust force was analyzed. The conclusions are summarized as follows:

1. As the depth increased during the machining of a micro pin, the thrust force gradually increased. This was because it became difficult to evacuate graphite chips as the depth increased.
2. As the tool rotation speed increased from 1500 to 3000 and 4500 rpm, the thrust force decreased by 57% and 66%. The thrust force showed an unstable increase at a rotational speed of 1500 rpm (feed per revolution: 400 nm/rev). Therefore, it is necessary to machine using a rotational speed over 3000 rpm.
3. The surface roughness of the bottom of the tool increased as the capacitance increased. When the capacitance was 500 nF, the surface roughness (Rt) was 9.317 µm and the grinding force decreased by 30% compared to the capacitance of 50 nF. Therefore, it is advantageous to use rough-surface tools with high capacitance.
4. When the number of machined micro pins increased to 5, the thrust force increased from 0.2 N to 1 N due to wear of the tool bottom. When the number of machined pins was 15, the surface roughness decreased by 14.95%, and when the number of machined pins was 900, the surface roughness of the tool bottom decreased by 82.48%.
5. To prevent tool breakage caused by the increasing of thrust force, EDM dressing was conducted to generate discharge craters on the worn surface. The dressed tool significantly reduced the force, allowing the machining of 900 pins using a single eccentric tool.

Because eccentric tools are fabricated by EDM, various conductive materials can be used as tool materials. In this study, tungsten carbide was used as the tool material, but polycrystalline diamond or polycrystalline cubic boron nitride (PCBN), which have much higher hardness, can also be used. Eccentric tools made of high hardness materials can be applied to the micro machining of other difficult-to-cut materials such as glass, ceramic, and tungsten carbide.

## Figures and Tables

**Table 1:** Machining parameters for micro eccentric tool.

Parameters	Value
Voltage (V)	100
Rotational speed (rpm)	2500
Feedrate (µm/s)	5
Capacitance (pF)	20,000 (Finish)
Dielectric fluid	Kerosene

**Table 2:** Machining parameters for micro pin machining.

Conditions	Value
Workpiece	Graphite
Feedrate (µm/s)	5, 10, 15
Rotational speed (rpm)	1500, 3000, 4500
Surface roughness of tool bottom (Ra)	0.628, 1.975, 2.713 µm
EDM dressing condition	100 V, 300 nF
Cutting oil	Kerosene

**Table 3:** EDM dressing conditions.

Conditions	Value
Workpiece	Graphite
Feedrate (µm/s)	5
Machining depth (µm)	5
Machining time (s)	2.3
Rotational speed (rpm)	3000
EDM dressing condition	100 V, 300 nF
Cutting oil	Kerosene

## Data Availability

Data are contained within the article.
